# Femoral Head Dislocation into the Rectum Following Blunt Trauma

**DOI:** 10.7759/cureus.4596

**Published:** 2019-05-03

**Authors:** Peter A Ebeling, Clarence Clark, Dylan Erwin, Katherine Beale, Daniel L Dent

**Affiliations:** 1 Surgery, University of Texas Health Science Center at San Antonio, San Antonio, USA; 2 Surgery, Morehouse School of Medicine and Grady Memorial Hospital, Atlanta, USA

**Keywords:** femoral head dislocation, rectal trauma, femur fracture, motor vehicle collision

## Abstract

Traumatic hip dislocations require prompt diagnosis and treatment to prevent avascular necrosis of the femoral head. This injury is further complicated when there is an ipsilateral femur fracture. Here, we present what is likely the first reported case of a patient with traumatic hip dislocation and ipsilateral femur fracture with transrectal displacement of the femoral head. The patient presented to a level one trauma center in 2006 as a transfer from another facility after being thrown from a pickup truck. Upon initial evaluation, a foreign body was palpated in the rectum. Computed tomography (CT) imaging showed that the right femoral head was lodged within the pelvis. In the operating room, an exploratory laparotomy was performed, and anoscopy confirmed the placement of the femoral head within the rectal lumen. The femoral head was extracted from the rectum transanally. The operation was abbreviated, as the patient became hemodynamically unstable, and he was taken to the intensive care unit. He returned to the operating room the following day for a repeat washout and proximal diversion. Despite numerous orthopedic procedures and operative washouts, he ultimately underwent a right hip disarticulation. Physicians should be aware that intracorporeal femoral head displacement is possible in select patients who have experienced a high-energy trauma mechanism. This is a complicated, highly morbid injury that poses various management challenges to orthopedic and acute care surgeons.

## Introduction

Femoral neck fractures are common injuries seen by orthopedic and acute care surgeons. In the elderly, they are commonly due to minor trauma and are more prevalent in women, while in young adults, these fractures are often associated with high-speed motor vehicle collisions [1]. The hip joint is a synovial ball and socket with significant stability, therefore, fractures and dislocations in young patients require significant force [2]. These injuries are often accompanied by complications that make treatment challenging. It is possible during high-energy traumatic mechanisms for the femoral neck to fracture completely and for the femoral head to lodge in ectopic positions. However, traumatic hip dislocation with concurrent femoral neck fracture is uncommon. Here, we present what may be the first report of traumatic hip dislocation with ipsilateral femoral neck fracture and transrectal displacement of the femoral head.

## Case presentation

In 2006, a 38-year-old man was ejected from the bed of a pickup truck and transferred to a level one trauma center for the evaluation and treatment of a complex pelvic fracture. A suprapubic catheter was placed prior to transfer due to a suspected severe urethral injury. The patient had no significant past medical history.

**Figure 1:**
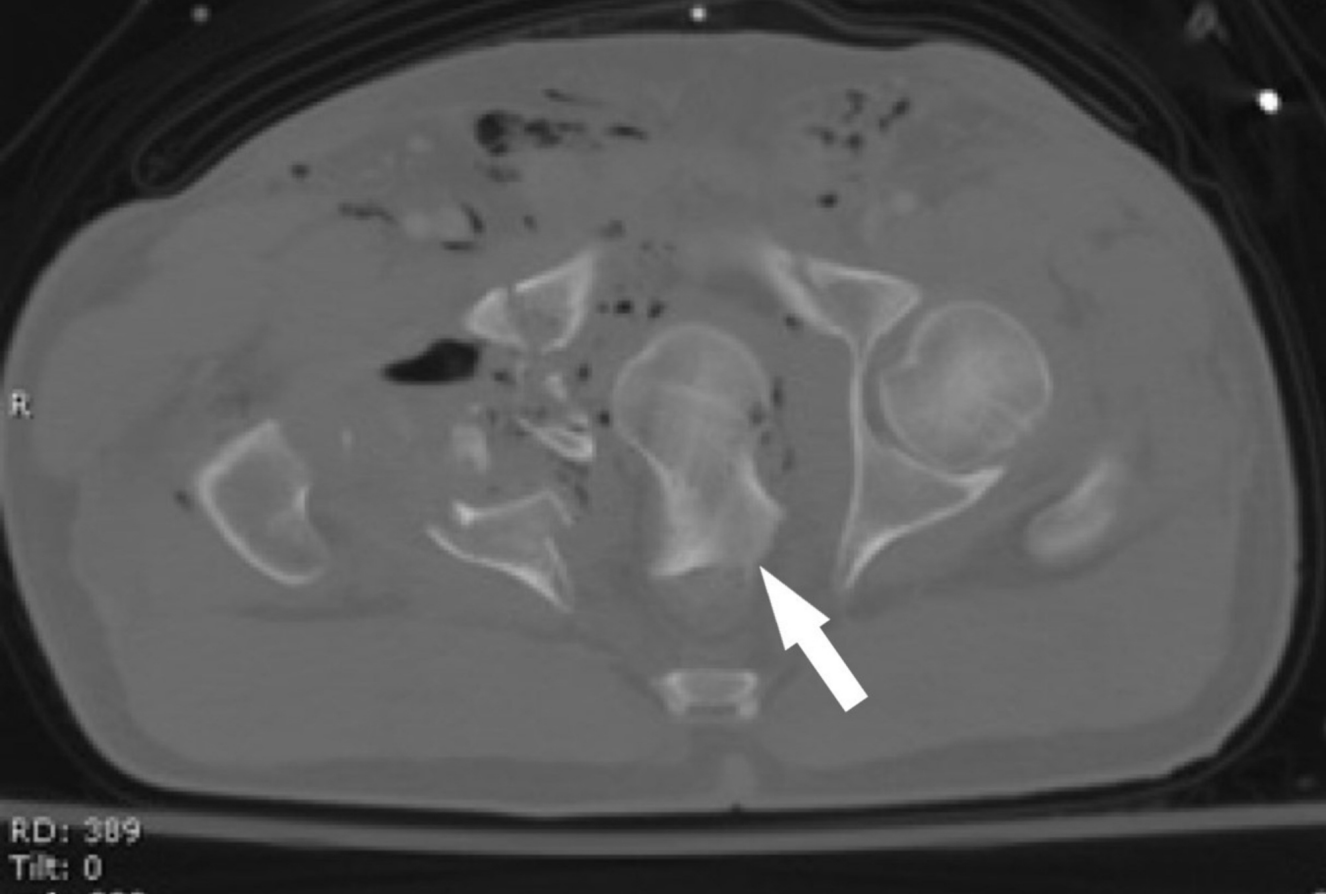
Axial plane CT imaging in the bone window setting showing the right femoral head (white arrow) penetrating the right acetabulum and right lateral wall of the rectum. CT: Computed tomography.

On arrival, the patient was tachycardic but normotensive and oxygenating adequately on room air. The physical exam was pertinent for an unstable pelvis and a digital rectal exam that revealed a foreign body in the rectal vault. There was normal rectal tone. The remainder of the exam revealed lacerations to the bilateral lower extremities with ecchymoses to the bilateral hips. Computed tomography (CT) evaluation revealed an intraperitoneal bladder rupture, a complex pelvic fracture involving the right sacroiliac joint, a shattered right acetabulum, a complete right femoral neck fracture, and the femoral head lodged in the pelvis (Figure 1).

The patient was taken to the operating room for an exploratory laparotomy and anoscopy. Anoscopy confirmed the femoral head was within the rectal vault with severe disruption of the right lateral rectum. The femoral head was removed from the rectum transanally, and the rectum was thoroughly irrigated. The bladder was repaired. 

The rectum and perirectal space were noted to have significant hematoma and fecal contamination that communicated with the acetabular fracture. Intraoperatively, the patient became critically ill, and the procedure was abbreviated. A temporary abdominal closure was placed, and the patient was taken to the intensive care unit for resuscitation. The patient returned to the operating room in 24 hours for fecal diversion and further debridement, irrigation, and drainage of the right perirectal space. Despite daily operative washouts, right hip disarticulation was ultimately required to achieve adequate debridement and sepsis control. After prolonged hospitalization, the patient was transferred to a long-term care facility out of state, which was closer to his home.

## Discussion

Traumatic hip dislocations and femoral head displacement

Hip dislocations are divided into anterior and posterior dislocations, with the latter comprising 85% of all cases [3]. Anterior dislocations are further divided into superior (pubic) and inferior (obturator). The vast majority of these injuries are due to high-energy mechanisms such as motor vehicle collisions. Due to the nature of the trauma mechanism, concomitant injuries are common. In total, 15% of patients with traumatic hip dislocations also have an intra-abdominal injury and 21% have a thoracic injury [4]. Various classification schemes exist to categorize hip dislocations and proximal femur fractures, most notably the Thompson and Epstein classifications [5]. However, it is difficult to apply either classification to our case, as they do not consider patients with a concomitant femoral head displacement into a hollow viscus.

The traumatic displacement of the femoral head with the involvement of the rectum is extremely rare. As far as we are aware, there do not appear to be other documented cases in the current literature. However, the femoral fracture-dislocation pattern is well described [6]. Liebergall et al. described femur fractures with concurrent dislocation and acetabular fractures as floating hip injuries, in which the femoral head is fractured from the femur and “floating” [7]. Broadly speaking, these injuries may present via two mechanisms. The posterior injury pattern is commonly thought to result from the car dashboard impacting the knee, transmitting force along the femur to the acetabulum, and forcing the femoral head posteriorly with resultant acetabulum fracture and hip displacement. Then, as the force continues to transmit up the femur, the diaphysis fractures. In contrast, the central injury pattern results from a lateral force on the hip, resulting in a multi-column fracture of the pelvis and femoral head displacement against the pelvic wall. Interestingly, this latter injury pattern is usually seen in patients who fall from a great height, but the patient in this report was ejected from a motor vehicle. It is certainly possible the forces experienced during high-speed vehicular ejections are similar to those in a multi-story fall.

There are several reports of the femoral head and neck lodging in ectopic locations following traumatic anterior hip dislocation and ipsilateral femur fractures. Schicho and Riepl describe a patient who was involved in a motorcycle collision with the resultant displacement of the left femoral head and neck into the scrotum [8]. The femoral head and neck were extracted from the scrotum, and he underwent successful orthopedic fixation of the hip. The patient was ultimately able to walk without a cane and did not have urologic sequelae. Jalili et al. reported the case of a 17-year-old man who suffered femoral head displacement into the perineum after a motorcycle collision [9]. This patient's case was also notable for a splenic injury requiring splenectomy and anal sphincter injury requiring repair. He underwent open reduction and internal fixation of the hip fracture, but his postoperative course was complicated by a surgical site infection necessitating repeated washouts. The patient’s mobility was ultimately severely limited secondary to avascular necrosis and resorption of the femoral head. Lastly, there is one report of an anterior, inferior hip displacement into the pelvis with an ipsilateral femur fracture and extraperitoneal bladder injury in a 14-year-old boy struck by a motor vehicle [10]. He underwent open reduction and internal fixation of the femur fracture, and the femoral head was reduced back into the joint. One year after surgery, the patient had radiographic evidence of avascular necrosis and was limping.

Injury management recommendations

This patient’s traumatic hip injury was an obvious surgical emergency, but the rectal injury added another layer of complexity to his care. Traumatic rectal injuries may be intra- or extraperitoneal. Historically, proximal diversion with antibiotic therapy was the mainstay of treatment, with direct repair becoming more common in the post-Vietnam War era [11]. Adjunctive measures, such as rectal washout and pre-sacral drain placement, gained popularity in this era but remained controversial. A recent multi-center retrospective review including 785 patients with rectal injuries provides additional data regarding the management of these injuries [12]. In this series, extraperitoneal injuries were successfully managed with either direct repair, observation, or proximal diversion. Interestingly, patients who had a pre-sacral drain placed or a rectal washout experienced a three-fold higher abdominal complication rate (defined as an intraperitoneal/retroperitoneal abscess or fascial dehiscence) as compared to patients without these adjuncts. Patients with intraperitoneal rectal injuries were diverted in 62% of cases, but these patients were also more likely to develop intra-abdominal complications. The study concludes that diversion for intraperitoneal injuries may be safely excluded when direct repair or resection and anastomosis is performed. It should be noted that 28% of the rectal injuries in this study were grade one (rectal hematoma or partial thickness injury), which may have skewed the results in favor of less aggressive operative management.

Management guidelines from the Eastern Association for the Management of Trauma (EAST) provide further rectal trauma management points [13]. This group conducted a systematic review of the literature from 1900 through 2014 for studies pertaining to penetrating, non-destructive extraperitoneal rectal injuries. Through a meta-analysis of 18 relevant articles, EAST conditionally recommends proximal diversion for non-destructive extraperitoneal rectal injuries and conditionally recommends against placing a pre-sacral drain and performing a distal rectal washout. Patients who received either of these adjunct therapies had higher mortality rates than patients who did not receive them, but as the authors note, it is unclear if the interventions contributed directly to their mortality or if these patients succumbed to their overall injury burden. Notably, patients who did not have pre-sacral drains placed had 40% fewer infectious complications than patients with a pre-sacral drain.

Reducing morbidity and mortality in large femur fractures with severe intracorporeal trauma presents a major surgical challenge. Open hip dislocations may be complicated by infection and avascular necrosis of the femoral head; the latter occurring in 6%-40% of cases [14]. The health burden of these complications can be significant. We have been unable to find large studies examining the long-term outcomes of patients with femoral fractures with intracorporeal displacement of the femoral head. Overall, this is not surprising. This is a rare injury pattern, and the overall injury burden may be so substantial that these patients do not live long enough to record long-term outcomes. Early recognition and reduction of a dislocated hip followed by definitive surgical intervention for fractures are key. History and physical following the advanced trauma life support protocol is necessary, and plain radiographs of the pelvis are critical. The addition of a CT scan of the hip if a femoral neck fracture is present may also be useful. Obviously, closed reduction in our patient’s case would not be possible, and in cases like this, emergent exploration in the operating room by the trauma and orthopedic teams is necessary.

## Conclusions

As far as the authors are aware, this is the first reported case of traumatic hip dislocation with ipsilateral femur fracture resulting in the transrectal displacement of the femoral head. Physicians should be aware that intracorporeal femoral head displacement is possible in select patients who have experienced a high-energy trauma mechanism. This is a complicated, highly morbid injury that poses various management challenges to orthopedic and acute care surgeons. We recommend emergent orthopedic intervention for the hip injury. For the rectal injury, we recommend repair or resection and anastomosis for an intraperitoneal injury. Diverting these patients remains an option. For an extra-peritoneal rectal injury, we agree with the EAST group’s conditional recommendation for proximal diversion. The use of adjuncts such as pre-sacral drains and rectal washout are controversial and may contribute to increased morbidity and mortality. Observation of the rectal injury would likely not be prudent in situations similar to this report, given the destructive nature of the injury and surrounding tissue devitalization. These patients will need to be brought back to the operating room for successive washouts and debridement.
